# Long Term Exposure to Microwave Radiation in Children Due to COVID-19 Pandemic; a Carcinogen Challenge

**DOI:** 10.34172/jrhs.2020.36

**Published:** 2020-12-19

**Authors:** Vida Zaroushani, Farahnaz Khajehnasiri

**Affiliations:** ^1^Social Determinants of Health Research Center, Research Institute for Prevention of Non-Communicable Disease, Qazvin University of Medical Sciences, Qazvin, Iran; ^2^Department of Occupational Health Engineering, Faculty of Health, Qazvin University of Medical Sciences, Qazvin, Iran.; ^3^Department of community medicine, School of Medicine, Tehran University of Medical Sciences, Tehran, Iran

## Dear Editor-in-Chief

Coronavirus 2019 (COVID-19), as an acute respiratory syndrome (SARS-CoV-2), was first identified in Wuhan, China, and spread rapidly in the world, including Iran, so that on December 14, 2020 (07:01 GMT) the number of total cases was 1,108,269 and the number of total death was 52,196 in Iran ^1^.

The coronavirus epidemic has affected education systems in Iran as around the world that lead to the closure of face-to-face education and the replacement of e-learning in schools and universities. In Iran, virtual education for students through various methods such as scheduled programs through television, mobile education through social media; and in university, it is done through learning management systems, such as NAVID and VESTA ^2^.

During the current global crisis, virtual education and e-Learning have some advantages over traditional teaching methods including cost-effectiveness, regular updates, flexibility to time and place, accessibility to instructional, overcoming the circumstances of the current lockdown, and less shyness to ask and interact with students ^3^. It rapidly pivoted toward digital and remote learning and could promote safety, comfort, and a feeling of control and reduce the spread of coronavirus infection by implementing social distance.

 Virtual education can also reduce human communication, interaction, contact surface, personal protective equipment, disinfectants, medical equipment, health, and medical staff loss, health, and medical care and their costs. Finally, it could lead to reduced mortality and total infected cases from covid-19, while traditional training programs do not have these advantages.

In such circumstances, the use of smartphones/tablets in e-learning is necessary and unavoidable and has caused children to access smartphones at an early age and be exposed to excessive microwave radiation from mobile phones ^2^.

 On the other hand, due to the need to use the Internet to access social networks and the use of virtual education, children are exposed to higher frequency microwave radiation.

Previous studies have shown that long-term exposure to microwave radiation due to excessive use of mobile phone caused several numbers thermal and non-thermal effects, including, skin effects, infertility disorders, brain problem, stress, mental disorder, behavioral changes, nervous system disease and disorder, Visual disorder, ear damage, oxidative stress and cancer ^4^.

the International Agency for Research on Cancer (IARC) 2011 categorization of radiofrequency radiation (RFR) from mobile phones and other wireless devices as a possible human carcinogen (Group 2B) ^5^.

Federal Communications Commission in Working closely with Food and Drug Administration has adopted limits for safe exposure to radiofrequency (RF) energy from 100 kHz to 100 GHz as the Specific Absorption Rate (SAR), which is a measure of the amount of radiofrequency energy absorbed by the body when exposed through microwave radiation. The applicable limits depend upon the type of sources (e.g., whether a cell phone or a broadcast transmitting antenna). Based on, the threshold level is a Specific Absorption Rate (SAR) value of 4 watts per kilogram (4 W/kg) for the whole body exposure to microwave and a 1.6 W/kg, averaged over one gram of tissue, as the safe limit for a mobile phone user microwave ^6^.

 Therefore, children's long-term exposure to mobile microwaves during e-learning during COVID-19 is an important and indirect corona health challenge that should be considered by parents, health policymakers, and governments. Due to the uncertainty of the end of the corona crisis and the acceptance of many educational systems of virtual and e-learning, it is necessary to develop a scheduled health program to track the health status of children.

 Moreover, due to the proximity of the cell phone to children's brains compared to adults, the average RF exposure due to its use may be higher by a factor of 2 in the child's brain [5]. However, parents can also apply effective primary administrative control to reduce microwave exposure from a mobile phone such as a diet enriched with antioxidants to improve the Child's immune system, disconnect microwave radiation through airplane or offline mode after downloading the educational content, decreasing sidelong activities by mobile phones such as games and professional and non-professional applications and using the speaker or headset mode to place it at a distance from the head.

## Acknowledgments

 The author would like to thanks Mr. Jalal Hassani for his support of this study.

## Conflict of interest statement

 The authors declare that there is no conflict of interests to declare.

## Funding

 This work has not a funding source.
